# Procollagen C-Endopeptidase Enhancer 2 Secreted by Tonsil-Derived Mesenchymal Stem Cells Increases the Oxidative Burst of Promyelocytic HL-60 Cells

**DOI:** 10.3390/biology11020255

**Published:** 2022-02-07

**Authors:** Hee-Soo Yoon, Hee-Yeon Kim, Kyung-Ah Cho, Yu-Hee Kim, So-Youn Woo, Han-Su Kim, Jihee-Lee Kang, Kyung-Ha Ryu, Joo-Won Park

**Affiliations:** 1 Department of Biochemistry, College of Medicine, Ewha Womans University, Seoul 07084, Korea; gltn129@naver.com (H.-S.Y.); heeyeon1432@gmail.com (H.-Y.K.); 2 Department of Microbiology, College of Medicine, Ewha Womans University, Seoul 07084, Korea; kyungahcho@ewha.ac.kr (K.-A.C.); kimyuhee@ewha.ac.kr (Y.-H.K.); soyounwoo@ewha.ac.kr (S.-Y.W.); 3 Department of Otorhinolaryngology, College of Medicine, Ewha Womans University, Seoul 07985, Korea; sevent@ewha.ac.kr; 4 Department of Physiology, College of Medicine, Ewha Womans University, Seoul 07084, Korea; jihee@ewha.ac.kr; 5 Inflammation-Cancer Microenvironment Research Center, College of Medicine, Ewha Womans University, Seoul 07084, Korea; 6 Department of Pediatrics, College of Medicine, Ewha Womans University, Seoul 07084, Korea

**Keywords:** neutrophil, tonsil derived mesenchymal stem cell, procollagen C-endopeptidase enhancer 2, oxidative burst, donor variation

## Abstract

**Simple Summary:**

Tonsil-derived mesenchymal stem cells (TMSCs) improved the reactive oxygen species (ROS) production in human promyelocytic leukaemia cells (HL-60) differentiated into neutrophil-like cells (dHL-60). TMSC-induced enhancement of ROS generation in dHL-60 cells was different depending on the TMSC donor. Comparison of RNA-sequencing data between high and low potentiating TMSC groups for ROS generation in dHL-60 cells showed elevated expressions of four genes: secreted frizzled-related protein 4, mesenteric estrogen-dependent adipogenesis, microfibrillar associated protein 5, and procollagen C-endopeptidase enhancer 2 (*PCOLCE2*). Real-time PCR and Western blotting confirmed high levels of PCOLCE2 in the high potentiating TMSC group for ROS generation in dHL-60 cells. In addition, knockdown of *PCOLCE2* in TMSCs reduced the enhancing efficacy of TMSCs regarding ROS generation in dHL-60 cells. Finally, treatment of recombinant PCOLCE2 protein augmented ROS production in dHL-60 cells with concomitant increases of NADPH oxidase (NOX) 3, NOX4, NOX5, and dual oxidase 2. Taken together, this study showed that PCOLCE2 levels in TMSCs could be used to select TMSCs with the high potentiating ability for ROS generation in neutrophils, and both TMSCs and PCOLCE2 may have the potential to enhance a frontline defence by increasing the efficiency of ROS generation in neutrophils.

**Abstract:**

Reactive oxygen species (ROS) generated by neutrophils provide a frontline defence against invading pathogens. We investigated the supportive effect of tonsil-derived mesenchymal stem cells (TMSCs) on ROS generation from neutrophils using promyelocytic HL-60 cells. Methods: Differentiated HL-60 (dHL-60) cells were cocultured with TMSCs isolated from 25 independent donors, and ROS generation in dHL-60 cells was measured using luminescence. RNA sequencing and real-time PCR were performed to identify the candidate genes of TMSCs involved in augmenting the oxidative burst of dHL-60 cells. Transcriptome analysis of TMSCs derived from 25 independent donors revealed high levels of procollagen C-endopeptidase enhancer 2 (PCOLCE2) in TMSCs, which were highly effective in potentiating ROS generation in dHL-60 cells. In addition, *PCOLCE2* knockdown in TMSCs abrogated TMSC-induced enhancement of ROS production in dHL-60 cells, indicating that TMSCs increased the oxidative burst in dHL-60 cells via PCOLCE2. Furthermore, the direct addition of recombinant PCOLCE2 protein increased ROS production in dHL-60 cells. These results suggest that PCOLCE2 secreted by TMSCs may be used as a therapeutic candidate to enhance host defences by increasing neutrophil oxidative bursts. PCOLCE2 levels in TMSCs could be used as a marker to select TMSCs exhibiting high efficacy for enhancing neutrophil oxidative bursts.

## 1. Introduction

Neutrophils participate in immune-mediated defences against invading pathogens as both the first line of innate defence and as effectors of adaptive immunity [1]. Neutrophils capture and kill microorganisms using dynamic strategies such as phagocytosis, generation of neutrophil extracellular traps, secretion of granules, and formation of reactive oxygen species (ROS) [2]. ROS production, which is also known as oxidative burst, is especially essential for neutrophils to destroy and eliminate invading microorganisms, and defects in ROS production lead to the survival of bacteria and uncontrolled infection [1]. Serious infectious diseases resulting from neutrophil dysfunction have been a significant cause of morbidity and mortality worldwide. For example, fatal infections during chemotherapy-induced neutropenia are one of the major causes of chemotherapy-related deaths [3]. In addition, a major cause of deaths occurring within 30 days of allogeneic hematopoietic cell transplantation involves infections such as pneumonia and sepsis [4]. High early mortality after hematopoietic cell transplantation is especially associated with the long recovery time of neutrophils, which are important for immune response, leading to neutrophil fever and sepsis [4]. Furthermore, a defective innate immune response, including neutrophil dysfunction, has been reported to be a host susceptibility factor during bacterial infection in diabetes [5]. Considering the short lifespan of neutrophils, enhancement of neutrophil function, including optimal ROS formation, can be useful for the prophylaxis of serious infections related to morbidity and mortality.

Mesenchymal stem cells (MSCs) are nonhematopoietic, multipotent fibroblast-like cells exhibiting potency to differentiate into mesodermal lineages, including osteocytes, adipocytes, and chondrocytes [6]. MSCs display specific characteristic cell surface markers, which are positive for clusters of differentiation (CD) 73, CD90, CD105, and negative for CD34 and human leukocyte antigen-DR [6]. MSCs can be easily isolated from various tissues, including bone marrow, adipose tissue, amniotic fluid, and Wharton’s jelly (substantia gelatinea funiculi umbilicalis), and possess immunomodulatory capacity, which enables successful allogeneic transplantation [6,7]. Previous studies suggest that MSCs can also modulate a variety of neutrophil functions such as migration, phagocytosis, and ROS production [7,8,9]. For example, MSCs derived from the compact bone in mice enhanced the capacity of neutrophils to phagocytose bacteria in vitro and in vivo, as well as promoted bacterial clearance in a murine sepsis model [10]. Similarly, interleukin-1β secreted from human placenta-derived MSCs, but not from human bone marrow-derived MSCs (BMSCs), enhanced the functional capacity of neutrophils as assessed by CD11b expression, ROS production, and phagocytosis of *Klebsiella pneumoniae* [11]. In addition, adipose tissue-derived MSCs (AMSCs) promoted the viability of neutrophils by inhibiting apoptosis as well as enhancing respiratory bursts, which could potentially be mediated by increased expression of interferon-α, granulocyte colony-stimulating factor, and transforming growth factor-β in AMSCs [12]. However, the supportive effect of MSCs on ROS generation from neutrophils is still controversial. In contrast to the enhanced ROS production of neutrophils by MSCs [11,12], reduced ROS formation from neutrophils after contact with MSCs has also been reported [13,14]. AMSCs robustly reduced ROS generation from neutrophils in a concentration-dependent manner and attenuated tissue damage in a murine immune complex-mediated vasculitis model of unbalanced neutrophil activation [14]. ROS production from feline neutrophils was decreased upon coculture with BMSCs, but not with AMSCs, and conditioned medium (CM) of both BMSCs and AMSCs reduced ROS generation from neutrophils [13]. Therefore, the precise role of MSCs in neutrophil oxidative bursts still remains to be elucidated and can be various depending on MSC origin and donor.

Tonsil-derived MSCs (TMSCs) are fibroblast-like adherent cells isolated from abandoned human palatine tonsils during tonsillectomy. They exhibit characteristics of MSCs, including surface marker expression, multilineage differentiation potential, and low immunogenicity [15,16,17]. TMSCs have higher proliferation rates than AMSCs and BMSCs, possibly due to the relatively young age of donors, with an average age of less than 10 years [17]. In addition, TMSCs can successfully differentiate into mesodermal lineage (bone, cartilage, and fat) and also into endodermal (insulin- or parathyroid- or estrogen-secreting cells and hepatocytes) or ectodermal lineages (Schwann cells) [16,17,18,19]. Furthermore, the excellent immunomodulatory properties of TMSCs and TMSC-CM demonstrated by in vivo animal studies [20,21,22] make TMSCs an ideal candidate for clinical applications. Considering the advantages of TMSCs, we investigated the effects of TMSCs on neutrophil function, including oxidative burst and viability. Because donor-to-donor heterogeneity is a major obstacle for developing standardised cell therapies, we analysed 25 independent TMSCs and newly discovered procollagen C-proteinase enhancer 2 (PCOLCE2) as a stimulating factor of neutrophil ROS production.

## 2. Materials and Methods

### 2.1. Cell Culture

The experimental procedures were approved by the Institutional Review Board of Ewha Womans University Mokdong Hospital (Seoul, Korea, EUMC 2020-02-028). After written informed consent was obtained, TMSCs were isolated from human palatine tonsils extracted from 25 independent donors undergoing tonsillectomy at Ewha Womans University Mok-dong Hospital (Seoul, Korea), and were cultured in Dulbecco’s modified Eagle’s medium (DMEM, Welgene, Daegu, Korea) supplemented with 10% fetal bovine serum (FBS, Corning, Corning, NY, USA) and 1% penicillin/streptomycin (P/S, Gibco, Waltham, MA, USA). HL-60 cells, a promyelocytic cell line, were purchased from American Type Culture Collection and cultured in Iscove’s Modified Dulbecco’s Medium (Welgene) supplemented with 10% FBS and 1% P/S. Three vials of human BMSCs were purchased from PromoCell (Heidelberg, Germany), American Type Culture Collection (Manassas, VA, USA), and Severance Hospital Cell Therapy Center (Seoul, Korea), and cultured in DMEM supplemented with 10% FBS and 1% P/S. The medium was changed every 2–3 days, and cells of passages 5–10 were utilised in the present study.

### 2.2. Differentiation of HL-60 Cells into Granulocyte-Like Cells

To differentiate HL-60 cells into neutrophil-like cells, HL-60 cells were cultured in Iscove’s Modified Dulbecco’s Medium supplemented with 10% FBS, 1% P/S, and 0.8% N, N-Dimethylformamide (Sigma Aldrich, St. Louis, MO, USA) for 4 days. Differentiation was confirmed by flow cytometry analysis, and differentiated HL-60 (dHL-60) cells were used to model human neutrophils [23].

### 2.3. Coculture of MSCs and dHL-60 Cells

TMSCs were seeded at a density of 5 × 10^5^ cells in 100 mm cell culture dishes, and then dHL-60 cells (1 × 10^6^ cells) were added dropwise onto preseeded TMSCs or BMSCs. Cocultured cells were maintained in DMEM supplemented with 10% FBS and 1% P/S. As a control group, only dHL-60 cells (1 × 10^6^ cells) were seeded in DMEM supplemented with 10% FBS and 1% P/S. Cocultured cells were incubated at 37 °C for 24 h. To harvest dHL-60 cells after coculture with MSCs, only the floating cells were carefully harvested, and Giemsa staining was performed according to the manufacturer’s protocol (Abcam, Cambridge, UK) to visualise the cells under microscopy.

### 2.4. Measurement of ROS

After dHL-60 cells were carefully harvested and washed twice with Hanks’ Balanced Salt Solution (Gibco), cells were resuspended in 100 μL of Hanks’ Balanced Salt Solution and seeded at a density of 1 × 10^5^ cells/well in a 96-well white plate. Then, the cells were stimulated with phorbol 12-myristate 13-acetate (PMA, 100 nM, Sigma Aldrich) for 15 min, and ROS was detected with a luminometer (Glomax Luminometer; Promega, Madison, WI, USA) after adding luminol (300 nM, Sigma Aldrich). To examine the effect of PCOLCE2 on ROS generation in dHL-60 cells, recombinant PCOLCE2 protein (Cusabio, Houston, TX, USA) was added with the indicated concentration for 24 h, and ROS was detected as mentioned above.

### 2.5. Collection of Conditioned Medium (CM)

TMSCs, cultured in 100 mm plates until 85% confluence, were washed three times with phosphate-buffered saline and incubated in serum-free media for 48 h. Then, the supernatant was collected and concentrated 50-fold by centrifugal filtration at 4 °C (Amicon Ultra-15 3 kDa filters; Millipore, Billerica, MA, USA).

### 2.6. Western Blotting

TMSCs were lysed in RIPA buffer (50 mM Tris–Cl [pH 7.5], 150 mM NaCl, 1% Nonidet P-40, 0.5% sodium deoxycholate, 0.1% sodium dodecyl sulfate) containing 50 mM NaF, 2 mM Na_3_VO_4_, and protease inhibitors (Sigma Aldrich). Proteins and CM samples, separated by sodium dodecyl sulfate-polyacrylamide gel electrophoresis, were transferred to PVDF membranes (Millipore, USA). Proteins transferred to membranes were confirmed with Ponceau S staining (Sigma Aldrich). After blocking membranes with 5% bovine serum albumin (BSA) in phosphate-buffered saline containing 0.1% Tween-20, membranes were subsequently incubated with primary antibodies; anti-PCOLCE2 (MyBioSource, San Diego, CA, USA) and anti α-tubulin (Sigma Aldrich). After washing, membranes were probed with horseradish peroxidase-conjugated secondary antibodies (Jackson ImmunoResearch Laboratories, West Grove, PA, USA). Following the chemiluminescent reaction, bands were identified with a Luminescent Image Analyzer LAS-4000 (Fuji, Tokyo, Japan). Bands were quantified using ImageJ (National Institute of Health, Bethesda, MD, USA).

### 2.7. Real-Time Polymerase Chain Reaction (PCR)

Total mRNA was extracted from cells with the RNeasy Mini Kit (Qiagen, Valencia, CA, USA) according to the manufacturer’s protocol. Complementary DNA (cDNA) was synthesised using a ReverTraAce qPCR RT master mix with a gDNA remover Kit (Toyobo, Osaka, Japan). Real time PCR amplification was conducted using the SYBR Green PCR Master Mix (Applied Biosystems, Foster City, CA, USA) in the ABI PRISM7500 sequence detection system (Applied Biosystems). The calculation of relative gene expression was carried out as 2^−ΔΔCt^. Primers are listed in Table S1.

### 2.8. Transfection of siRNA

TMSCs were transfected with siRNA for PCOLCE2 (50 nM, Invitrogen, Carlsbad, CA, USA) using Metafectene reagents (Biontex Laboratories GmbH, Munich, Germany). After 72 h, cells were utilised for further study. The sequences of predesigned Stealth Select siRNA (Thermo Fisher Scientific, Waltham, MA, USA) against PCOLCE2 are as follows: sense, 5′-UGGUGAUAGUCCACCUGCGCCAAUU-3′; antisense, 5′-AAUUGGCGCAGGUGGACUAUCACCA-3′.

### 2.9. Measurement of Viability of HL-60 Cells

After TMSCs or BMSCs were seeded at 5 × 10^4^ cells/well in a 96-well plate, dHL-60 cells were added at 1 × 10^5^ cells/well onto preseeded TMSCs or BMSCs. For PCOLCE2 treatment, recombinant PCOLCE2 protein (300 ng/mL) was added in dHL-60 cells. After 24 h of incubation, nonadherent dHL-60 cells were carefully transferred to a new 96-well plate. Purity of transferred cells was confirmed by Giemsa staining. Then, MTT solution (0.5 mg/mL) was added to each well, and cells were incubated for 4 h. After centrifugation at 400× *g* for 5 min, the medium was carefully removed. Then, 200 µL of DMSO was added to each well and absorbance was measured at 570 nm using a plate reader, Synergy H1M (Molecular Devices, Sunnyvale, CA, USA).

### 2.10. RNA Sequencing (RNA-seq)

Transcriptome analysis was performed using total RNA extracted from TMSCs. The library was prepared using the Illumina TruSeq Stranded mRNA Sample Prep Kit (Illumina, San Diego, CA, USA), and indexed libraries were then submitted to an Illumina NovaSeq (Illumina), and the paired-end (2 × 100 bp) sequencing was performed by the Macrogen (Seoul, Korea).

The reads were analysed as described previously [24]. Briefly, the raw reads were preprocessed from the sequencer to remove low quality and adapter sequences before analysis, and the processed reads were aligned to the *Homo sapiens* (*hg19*) using HISAT v2.1.0 [25]. Then, transcript assembly of known transcripts was processed by StringTie v1.3.4d [26], and the expression abundance of transcripts and genes were calculated as read counts, or Fragments Per Kilobase of exon per Million fragments mapped (FPKM) values. The expression profiles were used to conduct additional analysis, such as Differentially Expressed Genes (DEG).

### 2.11. Statistical Analysis of Gene Expression Levels in RNA-seq

Genes with an FPKM value of 0 in at least one sample were excluded from the analysis. After filtering, log2 based transformation was performed on the raw signal (FPKM) + 1 value for differential expression gene analysis. Data were corrected using quantile normalisation to reduce systematic bias that could affect biological meaning in intersample comparisons. Statistical significance of the differential expression data was evaluated using independent *t*-test and fold change in which the null hypothesis was that no difference exists among groups. The false discovery rate was controlled by adjusting the *p* value using the Benjamini–Hochberg algorithm. For the DEG set, hierarchical clustering analysis was carried out using complete linkage and Euclidean distance as a measure of similarity to display the expression patterns of differentially expressed transcripts, which were satisfied with |fold change| ≥ 2 and raw *p* value < 0.05. Enrichment of gene ontology analysis was performed for DEGs using g: Profiler [27].

### 2.12. Statistical Analysis

Values were presented as mean ± standard error of the mean (S.E.M.). Statistical significance was analysed using the Student’s *t* test or one-way analysis of variance using GraphPad PRISM 7 statistical software (GraphPad Software). A *p* value < 0.05 was considered statistically significant.

## 3. Results

### 3.1. TMSCs Augment ROS Generation from Differentiated HL-60

**Figure 1:**
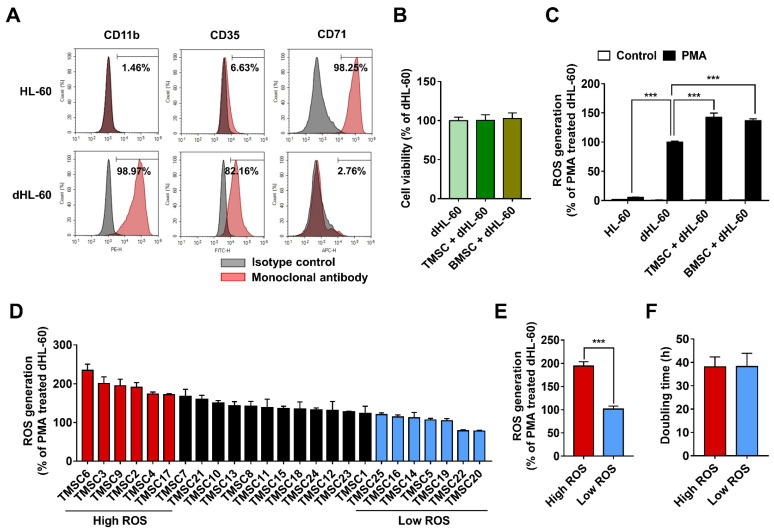
Tonsil-derived mesenchymal stem cells (TMSCs) potentiate reactive oxygen species (ROS) generation from differentiated HL-60 cells (dHL-60). (**A**) The levels of differentiation markers, CD11b, CD35, and CD71, measured by flow cytometry analysis. (**B**) The viability of dHL-60 cells measured after coculture with TMSCs or bone marrow-derived mesenchymal stem cells (BMSCs) for 48 h. (**C**) ROS generation from phorbol 12-myristate 13-acetate (PMA)-stimulated dHL-60 cells cocultured with TMSCs and BMSCs. (**D**) Donor variation of TMSCs in the potentiating capacity of ROS generation from dHL-60 cells. (**E**) The average ROS generation and (**F**) doubling time of high and low ROS groups. Values are expressed as the mean ± SEM (*n* = 3–25, *** *p* < 0.001).

To investigate the effects of TMSCs on neutrophil function, including oxidative burst and viability, TMSCs were cocultured with dHL-60 cells for 48 h. Because 6–12 × 10^6^ primary neutrophils that can be acquired from one mouse [28] are not sufficient for coculture assays with 25 TMSCs, dHL-60 cells were utilised as a neutrophil model [28,29]. dHL-60 cells share similarities with native neutrophils and are especially useful when a long-term culture or a large number of cells are required for study [30]. Differentiation of HL-60 into granulocyte-like cells was successfully induced with 0.8% N, N-Dimethylformamide, as previously reported [12,31]. Cell surface expression of granulocyte differentiation markers, CD11b and CD35, increased to 98.97% and 82.16%, respectively, on day four of differentiation (Figure 1A). Conversely, CD71, a marker of an erythroid lineage [32], significantly decreased to 2.76% on day four, compared with 98.25% on day 0 (Figure 1A). Based on these results, dHL-60 cells on differentiation day 4 were used as a neutrophil model for the current study. Considering that AMSCs improved viability and ROS generation of dHL-60 [12], we first examined whether TMSCs or BMSCs could improve the viability of dHL-60. The floating dHL-60 cells were carefully harvested after coculture with MSCs, and the percentage of dHL-60 cells in floating cells after the coculture with MSCs was 98.62 ± 0.94% as assessed by Giemsa staining (Figure S1). Neither TMSCs nor BMSCs altered the viability of dHL-60 (Figure 1B). Next, ROS generation from dHL-60 was evaluated upon coculture with TMSCs or BMSCs, and PMA, a pharmacological NADPH oxidase (NOX) activator [33], was used to stimulate ROS production from dHL-60 cells. As expected, PMA stimulation caused an oxidative burst in dHL-60 cells, and both TMSCs and BMSCs similarly increased ROS generation from dHL-60 cells (Figure 1C).

To examine whether donor variation, which needs to be overcome to develop standard cell therapies, existed in TMSCs, TMSCs isolated from 25 independent donors were evaluated. Although most TMSCs enhanced ROS generation from PMA-stimulated dHL-60 cells, the degree of enhancement was different depending on the donors (Figure 1D). To analyse the difference among TMSCs with a variety of supportive capacities for ROS generation from PMA-stimulated dHL-60 cells, TMSCs were distributed in high ROS and low ROS groups. High and low ROS groups included TMSCs, which potentiated ROS generation from PMA-stimulated dHL-60 cells at greater than 70% and less than 20%, respectively (Figure 1D). The average ROS produced from dHL-60 cells was 194.4 ± 9.3% in the high ROS group and 101.8 ± 6.5% in the low ROS group, compared with the PMA-only-stimulated group (Figure 1E). The difference of ROS generations from dHL-60 cells between the high and low ROS groups was statistically significant (Figure 1E). However, no significant difference in doubling time was observed between the two groups (Figure 1F).

### 3.2. RNA-Sequencing Data Analysis of TMSCs

**Figure 2:**
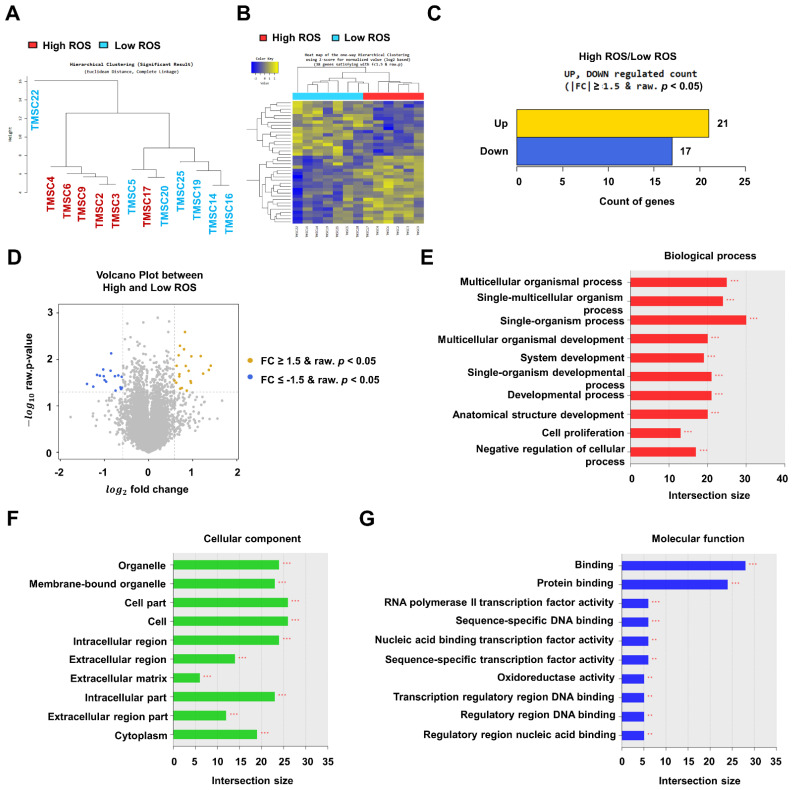
RNA-sequencing data analysis of tonsil-derived mesenchymal stem cells (TMSCs) distributed in the high and low reactive oxygen species (ROS) groups. (**A**) A dendrogram of hierarchical clustering, (**B**) a heat map, (**C**) genes significantly up- and downregulated by more than 1.5-fold, and (**D**) a volcano plot between the high and low ROS groups. The Gene Ontology analysis of (**E**) biological processes, (**F**) cellular component, and (**G**) molecular function (** *p* < 0.01; *** *p* < 0.001).

RNA-sequencing (RNA-seq) data of 25 TMSCs acquired to analyse the donor variation were taken from Kim et al. (*submitted*). To examine whether the gene expression pattern was different between the high and low ROS groups, TMSCs of each group were compared by RNA-seq. To confirm the difference between the two TMSC groups, RNA-seq data of TMSCs were analysed using Pearson’s correlation coefficient and unsupervised hierarchical clustering (Figure 2A). Except for TMSC17 and TMSC22, TMSCs in each group were clustered together (Figure 2A). A heat map showed the distinct gene expression patterns of each group (Figure 2B). The genes altered by more than 1.5-fold between groups are shown in Figure 2C. More specifically, upregulated genes with the threshold set to “*p* < 0.05, fold change > 1.5” in the high ROS group were 21 when compared with the low ROS group (Table 1). Downregulated genes with the threshold set to “*p* < 0.05, fold change < −2” in the high ROS group were 17 when compared with the low ROS group (Table 2). A volcano plot shows the 38 significantly altered genes (Figure 2D). Gene-set enrichment analysis was performed based on the Gene Ontology (GO) database of the high ROS group compared with the low ROS group. Three categories of GO, including biological process, cellular component, and molecular function, were analysed (Figure 2E–G). Organismal and developmental processes were increased in the biological process (Figure 2F), and the binding function, including protein binding, was selected in the molecular process (Figure 2G).

### 3.3. PCOLCE2 mRNA and Protein Levels Are Elevated in the High ROS Group when Compared with the Low ROS Group

**Figure 3:**
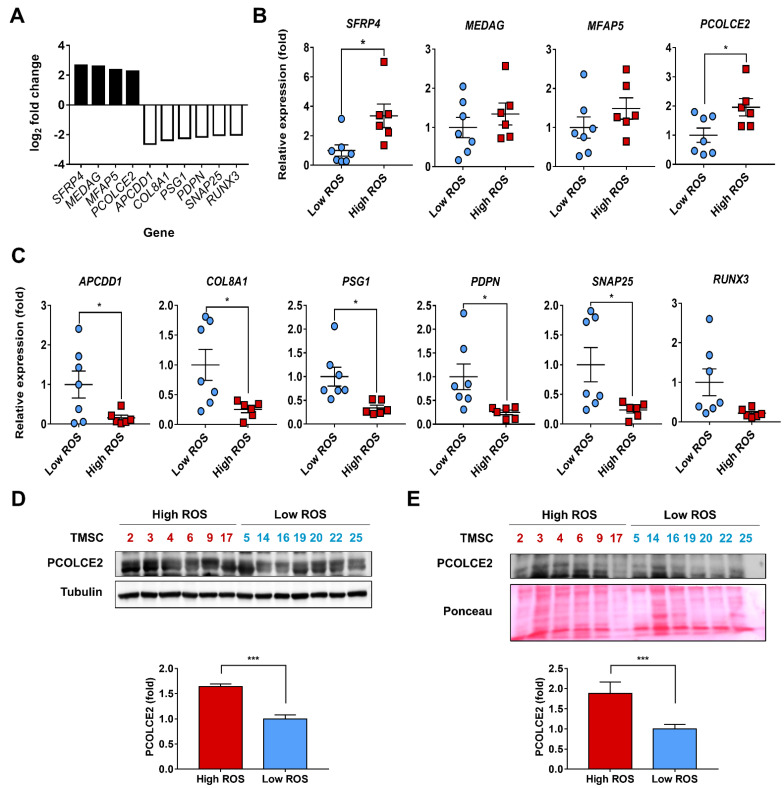
PCOLCE2 expression is increased in the high reactive oxygen species (ROS) group compared with the low ROS group. (**A**) Altered genes by RNA sequencing more than 2-fold in the high ROS group compared with the low ROS group. The (**B**) increased and (**C**) decreased genes in the high ROS group were confirmed using real-time PCR. The protein levels of PCOLCE2 were analysed with (**D**) cell lysates and (**E**) conditioned medium using Western blotting and quantified with ImageJ (Figure S2). Values are expressed as the mean ± SEM (*n* = 6−7; * *p* < 0.05; *** *p* < 0.001). APCDD1—adenomatosis polyposis downregulated 1; COL8A1—collagen type VIII alpha 1 chain; MEDAG—mesenteric estrogen-dependent adipogenesis; MFAP5—microfibrillar associated protein 5; PCOLCE2—procollagen C-endopeptidase enhancer 2; PDPN—podoplanin; PSG1—pregnancy-specific beta-1-glycoprotein 1; RUNX3—runt related transcription factor 3; SFRP4—Secreted frizzled-related protein 4; SNAP25—synaptosome associated protein 25.

RNA sequencing revealed ten genes that were increased and decreased more than 2-fold in the high ROS group compared with the low ROS group (Figure 3A). Genes increased more than 2-fold in the high ROS group included secreted frizzled-related protein 4 (*SFRP4*), mesenteric estrogen-dependent adipogenesis (*MEDAG*), microfibrillar associated protein 5 (*MFAP5*), and procollagen C-endopeptidase enhancer 2 (*PCOLCE2*). Genes decreased more than 2-fold were adenomatosis polyposis downregulated 1 (*APCDD1*), collagen type VIII alpha 1 chain (*COL8A1*), pregnancy-specific beta-1-glycoprotein 1 (*PSG1*), podoplanin (*PDPN*), synaptosome-associated protein 25 (*SNAP25*), and runt-related transcription factor 3 (*RUNX3*). We further confirmed the alterations of these genes using real-time PCR. Levels of two genes, *SFRP4* and *PCOLCE2*, were significantly elevated (Figure 3B), and the decreases of five genes, *APCDD1*, *COL8A1*, *PSG1*, *PDPN*, and *SNAP25*, were statistically significant (Figure 3C). The potentiating role of TMSCs on ROS production from dHL-60 cells can be derived from paracrine signalling or direct cell-to-cell contact. Because both SFRP4 and PCOLCE2 can be secreted, these were candidates responsible for the paracrine effects of TMSCs. Wnt signalling has been reported to activate NOX4 and ROS generation [34], and SRFP4 is an antagonist of the Wnt pathway [35]. We, therefore, further focused on *PCOLCE2*, rather than *SFRP4*, as a candidate gene for potentiating ROS generation from dHL-60 cells. Elevated PCOLCE2 protein levels in the high ROS group were confirmed by Western blotting (Figure 3D). PCOLCE2 protein expression was 1.6 ± 0.1-fold higher in the high ROS group when compared with the low ROS group (Figure 3D). In addition, secreted PCOLCE2 protein levels in TMSC-CM were 1.88 ± 0.28-fold higher in the high ROS group compared with the low ROS group (Figure 3E).

### 3.4. Neutrophil ROS Production Is Enhanced by PCOLCE2

**Figure 4:**
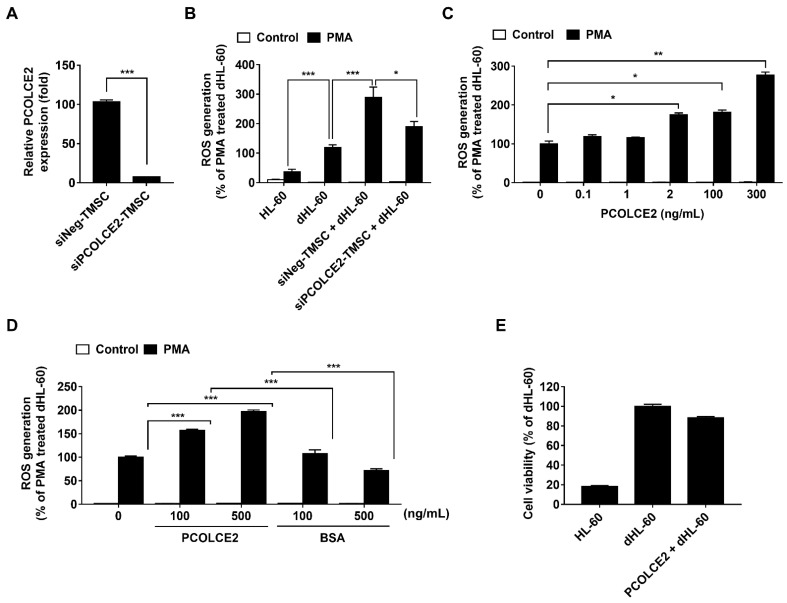
PCOLCE2 promotes reactive oxygen species (ROS) generation from dHL-60 cells. (**A**) The siRNA-mediated *PCOLCE2* knockdown confirmed with real-time PCR. (**B**) ROS generation from dHL-60 cells after (**B**) coculture with *PCOLCE2* knockdown tonsil-derived mesenchymal stem cells (TMSCs) or (**C**) addition of PCOLCE2 protein. (**D**) ROS generation from dHL-60 cells treated with PCOLCE2 protein or bovine serum albumin (BSA). (**E**) The cell viability after PCOLCE2 treatment. Values are expressed as the mean ± SEM (*n* = 5; * *p* < 0.05; ** *p* < 0.01; *** *p* < 0.001). The siNeg-TMSC—TMSCs treated with siRNA for negative control; siPCOLCE2-TMSC—TMSCs transfected with siRNA for *PCOLCE2*.

To investigate whether elevated PCOLCE2 in the high ROS group played a key role for TMSCs in potentiating ROS generation from dHL-60 cells, siRNA-mediated gene knockdown was performed. Transfection of siRNA targeted to *PCOLCE2* successfully downregulated *PCOLCE2* expression (Figure 4A), and *PCOLCE2* gene knockdown abrogated the potentiating effect of TMSCs on PMA-stimulated ROS generation from dHL-60 cells (Figure 4B). In addition, treatment of recombinant PCOLCE2 protein with a concentration >2 ng/mL enhanced ROS production from dHL-60 cells in a dose-dependent manner (Figure 4C). To confirm that the potentiating effect of PCOLCE2 on ROS generation from dHL-60 cells was specific, BSA was treated with the same concentration of PCOLCE2. Unlike PCOLCE2, BSA did not promote an oxidative burst of dHL-60 cells, indicating a specific role of PCOLCE2 in promoting ROS production from dHL-60 cells (Figure 4D). We next evaluated whether PCOLCE2 also affected the viability of dHL-60 cells. Consistent with the results of Figure 1B, PCOLCE2 treatment did not alter the viability of dHL-60 cells (Figure 4E).

### 3.5. PCOLCE2 Alters the Levels of Genes Involved in Neutrophil Function

**Figure 5:**
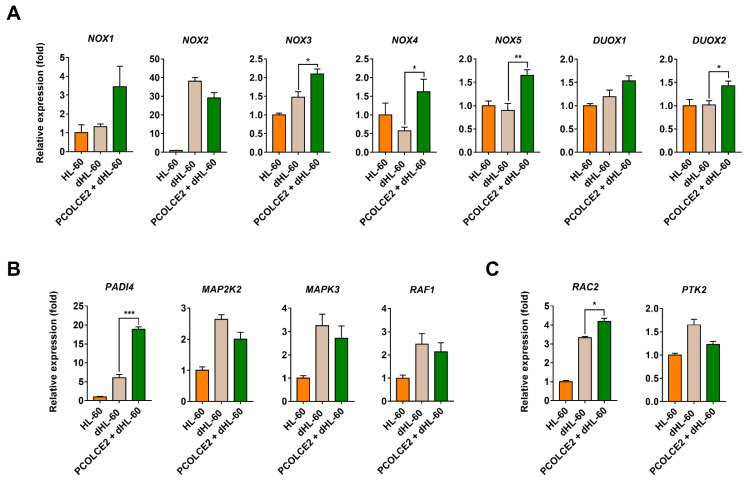
Alteration of genes involved in neutrophil function by PCOLCE2. Expression of genes involved in (**A**) oxidative burst, (**B**) formation of neutrophil extracellular traps, and (**C**) phagocytosis were analysed by real-time PCR. Values are expressed as mean ± S.E.M. (*n* = 4; * *p* < 0.05; ** *p* < 0.01; *** *p* < 0.001). DUOX—dual oxidase; MAP2K2—mitogen-activated protein kinase kinase 2; MAPK3—mitogen-activated protein kinase 3; NOX—NADPH oxidase; PADI4—peptidyl arginine deiminase 4; PTK2—protein tyrosine kinase 2; RAC2—Rac family small GTPase 2; RAF1—Raf-1 proto-oncogene, serine/threonine kinase.

To elucidate the molecular mechanism for PCOLCE2 enhancement of ROS generation from dHL-60 cells, genes involved in neutrophil function were examined using real-time PCR. Because NOX and dual oxidase (DUOX) mainly play an essential role in ROS generation, five isotypes of NOX and two isotypes of DUOX were examined. Among genes involved in the oxidative burst, NOX3, NOX4, NOX5, and DUOX2 mRNA levels were significantly increased by PCOLCE2 (Figure 5A). We further examined genes involved in the formation of neutrophil extracellular traps (NETs) and phagocytosis. PCOLCE2 also increased the expression of peptidyl arginine deiminase 4 (PADI4), which is critical for NET formation, and the Rac family small GTPase 2 (RAC2), which regulates phagocytosis (Figure 5B,C).

## 4. Discussion

TMSCs were noninvasively obtained from waste tissues during a tonsillectomy, which is one of the most frequently performed surgical procedures in children [17]. TMSCs exhibit superiority over other MSCs regarding proliferation rate, differentiation potency, and immunomodulatory capacity [16,17,36]. Because TMSCs successfully maintain MSC-specific properties during freezing and thawing, and also up to passage 15 during long-term in vitro cell culture [36], TMSCs are a good MSC candidate for clinical cell therapy. Our recent studies suggested that co-transplantation of bone marrow cells and TMSCs improved the outcomes of bone marrow transplantation by enhancing bone marrow cell engraftment [37] and thymus regeneration [38]. Because the infection is significantly associated with morbidity and mortality among patients receiving allogeneic bone marrow cells, enhancing neutrophil function is critical in maintaining host immune defences against invading microorganisms during bone marrow transplantation. The present study showed that cocultured TMSCs with dHL-60 enhanced ROS production in dHL-60, implicating the ability of TMSCs to promote an oxidative burst of neutrophils, which is essential in the clearance of invading pathogens.

Cell therapy has been regarded as a revolution in regenerative medicine, and MSCs are a promising and valid source of cell therapy. Because standardisation and monitoring of MSCs are prerequisites for reproducible clinical application, donor-dependent variations of MSCs should be controlled and overcome. The capacity of TMSCs in promoting oxidative bursts of dHL-60 cells depended on the donors. A similar donor variation was previously observed in umbilical cord blood MSCs in regards to cell proliferation and immune-modulatory functions [39]. In addition, BMSCs from three swine donors exhibited varied morphologies, growth rates, and doubling times, and the functional potency of the BMSCs on the inhibition of endothelial permeability was also cell donor-dependent [40]. RNA-seq analysis of TMSCs, grouped by the supportive capability of ROS generation from dHL-60 cells, identified intrinsic variations in gene expression profiles. In the high ROS generation group, genes including *sFRP4* and *PCOLCE2* were significantly increased, and protein binding was also upregulated, as assessed using GO analysis. Both sFRP4 and PCOLCE2 are secreted and bind to proteins. FRP4 is a secreted Wnt antagonist that directly binds to Wnts [41], and PCOLCE2 binds the COOH-terminal propeptide of type I procollagen and enhances the cleavage of procollagen, by procollagen C-proteinases, such as bone morphogenetic protein (BMP) 1 [42].

PCOLCE2 is an extracellular matrix glycoprotein playing a role of a functional procollagen C-proteinase enhancer [42]. PCOLCE2 treatment increased the expressions of NOX3, NOX4, NOX5, and DUOX2. NOX is a superoxide-generating enzyme, and ROS in neutrophils are mostly produced by NOX. Among NOX isoforms, NOX2 is mainly expressed in neutrophils and supplies bursts of O_2_^−^, which are critical for bactericidal responses [43]. However, NOX2 expression was not altered by PCOLCE2. Although NOX1, NOX3, NOX4, NOX5, DUOX1, and DUOX2 are usually expressed in a variety of nonphagocytic cells, they can definitely generate ROS [44]. Therefore, the combinational increase of NOX3, NOX4, NOX5, and DUOX2 may lead to increased ROS production by PCOLCE2 from dHL-60 cells. Notably, PCOLCE2 treatment significantly increased PADI4 expression. PADI4 is required for the extrusion of NETs and has an essential role in NET formation [45,46]. Because ROS generation is also required for PADI4 activation [46], ROS and NET formation can positively regulate each other. However, the functional roles of PCOLCE2 and TMSCs in other neutrophil functions such as NET formation and phagocytosis remain to be elucidated.

Reduced genes in the high ROS generation group involved *APCDD1*, *COL8A1*, *PSG1*, *PDPN*, and *SNAP25*. Because APCDD1 is a dual inhibitor of BMP and Wnt signalling pathways [47], reduction of APCCD1 may induce activation of BMP and Wnt signalling pathways. COL8A1 is a collagen type VIII protein, and upregulation of COL8A1 has been correlated with tumour cell proliferation and invasion [48]. PSG1 is a biological activator of TGF-β and reduces inflammation in a dextran sodium sulfate-induced murine colitis model [49]. PDPN is a mucin-type transmembrane protein used as a marker for lymphatic endothelial cells and fibroblastic reticular cells of lymphoid organs such as tonsils [50]. SNAP25 is a component of the SNARE complex and also regulates intracellular calcium levels by interacting with calcium channel proteins [51]. The comprehensive effects of these alterations in TMSCs remain to be examined.

In the current study, both TMSCs and BMSCs potentiated ROS generation in PMA-stimulated dHL-60 cells but did not affect dHL-60 cell viability. MSCs have been reported to alter neutrophil functions, including cell viability, respiratory bursts, and phagocytosis [7,8]. Both exosomes and CM of Wharton’s jelly MSCs increase neutrophil lifespan and phagocytosis, but only CM of Wharton’s jelly MSCs promotes neutrophil respiratory bursts [52]. AMSCs also enhance the viability of neutrophils and respiratory bursts [12]. Similarly, BMSCs inhibit apoptosis of resting and IL-8-stimulated neutrophils, which is mediated by secreted factor IL-6. However, BMSCs do not alter ROS production by neutrophils, which were isolated from healthy volunteers, in a PMA-stimulated condition [53]. The discrepancy among studies can be attributed either to the different cell sources or donor variations. Indeed, ROS production from dHL-60 cells cocultured with TMSCs from 25 independent donors was variable, reaffirming the importance of strategies to overcome donor variations. PCOLCE2 levels in TMSCs may select TMSCs exhibiting high efficacies for enhancing neutrophil oxidative bursts.

In summary, the present study demonstrated the potentiating capacity of TMSCs in neutrophil ROS generation and revealed PCOLCE2 as a novel enhancer of neutrophil oxidative bursts. PCOLCE2 could be used as a marker to select TMSCs for promoting neutrophil ROS production. Furthermore, TMSCs and PCOLCE2 may be clinically used as novel therapies to reduce morbidity and mortality associated with infection by potentiating neutrophil ROS formation.

## Figures and Tables

**Table 1:** Genes increased in the high ROS group compared with the low ROS group, more than 1.5-fold according to RNA-seq data.

Gene Number	Gene Symbol	Gene Full Name	High/Low Group (Fold)
NM_003014	*SFRP4*	Secreted frizzled-related protein 4	2.66
NM_032849	*MEDAG*	Mesenteric estrogen-dependent adipogenesis	2.58
NM_001297709	*MFAP5*	Microfibrillar-associated protein 5	2.36
NM_013363	*PCOLCE2*	Procollagen C-endopeptidase enhancer 2	2.27
NM_001162371	*LOC728392*	Uncharacterised LOC728392	1.96
NM_000908	*NPR3*	Natriuretic peptide receptor 3	1.95
NM_001309443	*SPARC*	Secreted protein acidic and cysteine-rich	1.93
NR_001591	*TPTEP1*	Transmembrane phosphatase with tensin homology pseudogene 1	1.88
NM_001039667	*ANGPTL4*	Angiopoietin-like 4	1.82
NM_001134851	*TCF7*	Transcription factor 7 (T-cell specific, HMG-box)	1.79
NM_001307952	*HAPLN3*	Hyaluronan and proteoglycan link protein 3	1.77
NM_006332	*IFI30*	IFI30, lysosomal thiol reductase	1.75
NM_000398	*CYB5R3*	Cytochrome b5 reductase 3	1.69
NM_001190737	*NFIB*	Nuclear factor I B	1.64
NM_178507	*OAF*	Out at first homolog	1.64
NM_006617	*NES*	Nestin	1.62
NM_000710	*BDKRB1*	Bradykinin receptor B1	1.62
NM_005251	*FOXC2*	Forkhead box C2 (MFH-1, mesenchyme forkhead 1)	1.60
NM_144617	*HSPB6*	Heat shock protein family B (small) member 6	1.56
NM_016932	*SIX2*	SIX homeobox 2	1.54
NM_173554	*C10orf107*	Chromosome 10 open reading frame 107	1.50

**Table 2:** Genes decreased in the high ROS group compared with the low ROS group, more than 1.5-fold according to RNA-seq data.

Gene Number	Gene Symbol	Gene Full Name	High/Low Group (Fold)
NM_153000	*APCDD1*	APC downregulated 1	−2.63
NM_001850	*COL8A1*	Collagen type VIII alpha 1 chain	−2.38
NM_001184825	*PSG1*	Pregnancy-specific beta-1-glycoprotein 1	−2.25
NM_001006624	*PDPN*	Podoplanin	−2.15
NM_001322902	*SNAP25*	Synaptosome associated protein 25	−2.04
NM_001031680	*RUNX3*	Runt-related transcription factor 3	−2.02
NM_001242702	*MKX*	Mohawk homeobox	−1.99
NM_000902	*MME*	Membrane metalloendopeptidase	−1.95
NM_001287426	*UPP1*	Uridine phosphorylase 1	−1.82
NM_016246	*HSD17B14*	Hydroxysteroid 17-beta dehydrogenase 14	−1.80
NM_133448	*TMEM132D*	Transmembrane protein 132D	−1.70
NM_001128166	*PAK3*	p21 (RAC1) activated kinase 3	−1.68
NM_001253835	*IGFBP7*	Insulin-like growth factor-binding protein 7	−1.61
NM_207510	*LCNL1*	Lipocalin like 1	−1.56
NM_002153	*HSD17B2*	Hydroxysteroid 17-beta dehydrogenase 2	−1.54
NM_000827	*GRIA1*	Glutamate ionotropic receptor AMPA type subunit 1	−1.52
NM_001002294	*FMO3*	Glutamate ionotropic receptor AMPA type subunit 1	−1.52

## Data Availability

The data presented in the present study are available on request from the corresponding authors.

## Supplementary Materials

The following supporting information can be downloaded at: https://www.mdpi.com/article/10.3390/biology11020255/s1, Table S1: Primers used in real-time PCR, Figure S1: The floating cells in the supernatant of cocultured tonsil-derived mesenchymal stem cells (TMSCs) and differentiated HL-60 (dHL-60) cells are mostly dHL-60 cells, Figure S2: Full Western Blot of Figure 3D,E.

## List of Abbreviations

AMSC	Adipose tissue-derived mesenchymal stem cell
APCDD1	Adenomatosis polyposis downregulated 1
BMP	Bone morphogenetic protein
BMSC	Bone marrow-derived mesenchymal stem cell
BSA	bovine serum albumin
CD	Clusters of differentiation
CM	Conditioned medium
COL8A1	Collagen type VIII alpha 1 chai
dHL-60	Differentiated HL-60
DUOX	Dual oxidase
DEG	Differentially expressed genes
DMF, MAPK3	Mitogen-activated protein kinase 3
FKRM	Fragments Per Kilobase of exon per Million fragments mapped
GO	Gene ontology
MAP2K2	Mitogen-activated protein kinase kinase 2
MEDAG	Mesenteric estrogen-dependent adipogenesis
MFAP5	Microfibrillar-associated protein 5
MSC	Mesenchymal stem cell
NET	Neutrophil extracellular trap
NOX	NADPH oxidase
PADI4	Peptidyl arginine deiminase 4
PCOLCE2	Procollagen C-endopeptidase enhancer 2
PCR	polymerase chain reaction
PDPN	Podoplanin
PMA	phorbol 12-myristate 13-acetate
PSG1	Pregnancy-specific beta-1-glycoprotein 1
PTK2	Protein tyrosine kinase 2
RAC2	Rac family small GTPase 2
RAF1	Raf-1 proto-oncogene serine/threonine- kinase
RNA-seq	RNA sequencing
ROS	Reactive oxygen species
RUNX3	Runt related transcription factor 3
SFRP4	Secreted frizzled-related protein 4
SNAP25	Synaptosome-associated protein 25
TMSC	Tonsil-derived mesenchymal stem cell
