# Supplementary material for: Procollagen C-Endopeptidase Enhancer 2 Secreted by Tonsil-Derived Mesenchymal Stem Cells Increases the Oxidative Burst of Promyelocytic HL-60 Cells
Source: Biology (Basel). 2022 Feb 7;11(2):255. doi: 10.3390/biology11020255 (PMC8869569; doi:10.3390/biology11020255)
Supplement: Supplementary file 1 [file biology-11-00255-s001.zip › biology-1550657-supplementary.pdf]

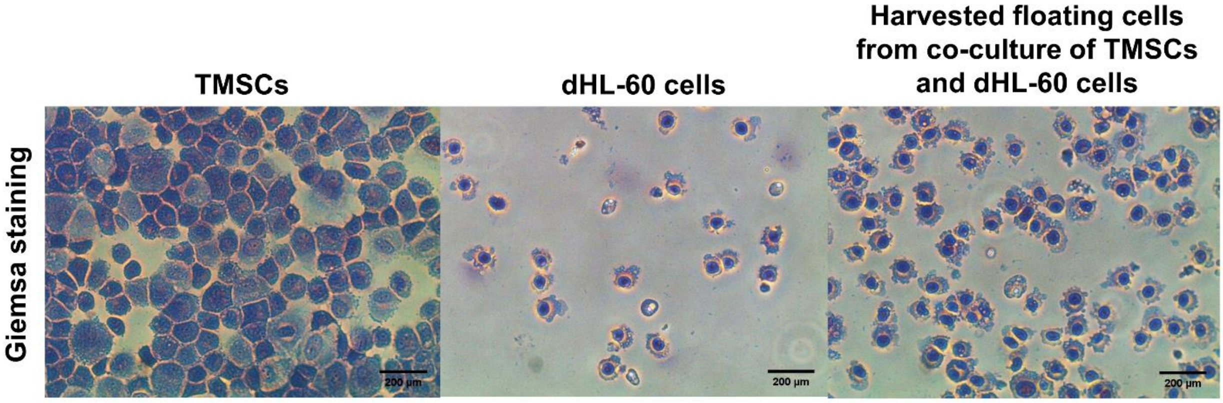

Supplementary Figure S1. The floating cells in the supernatant of co-cultured tonsil-derived mesenchymal stem cells (TMSCs) and differentiated HL-60 (dHL-60) cells are mostly dHL-60 cells. Giemsa staining was shown to demonstrate the morphology of the harvested floating cells from the co-culture of TMSCs and dHL-60 cells. Image magnification, 200×. Scale bars, 200 µm.

A. PCOLCE2

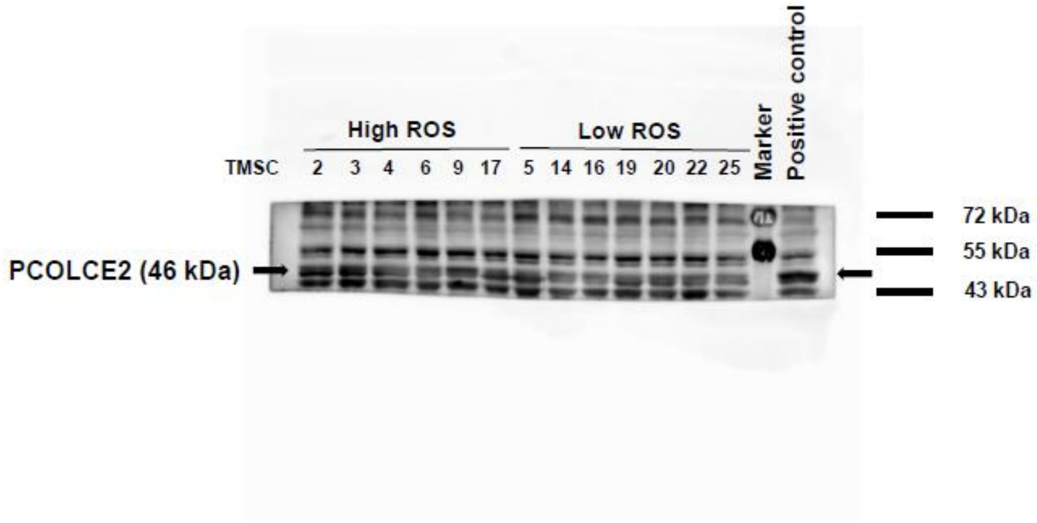

B. Tubulin

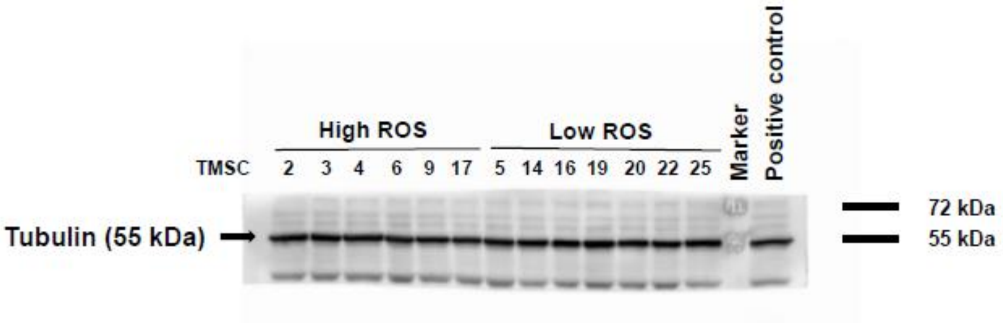

C. PCOLCE2

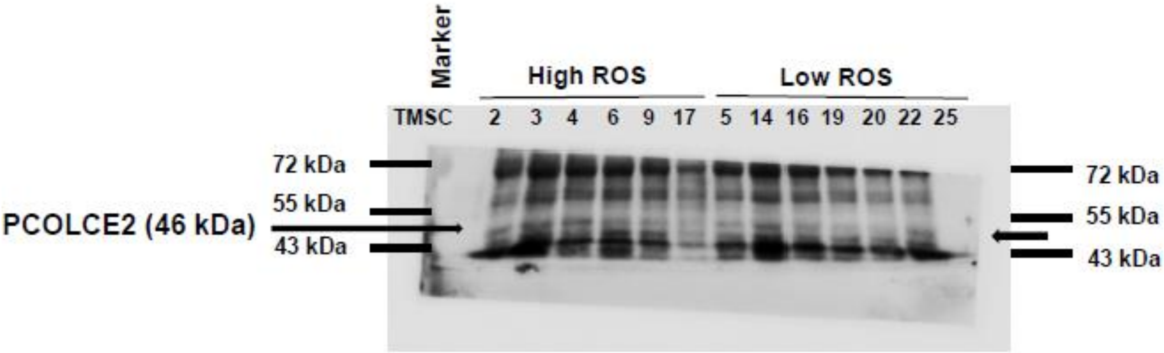

D. Ponceau

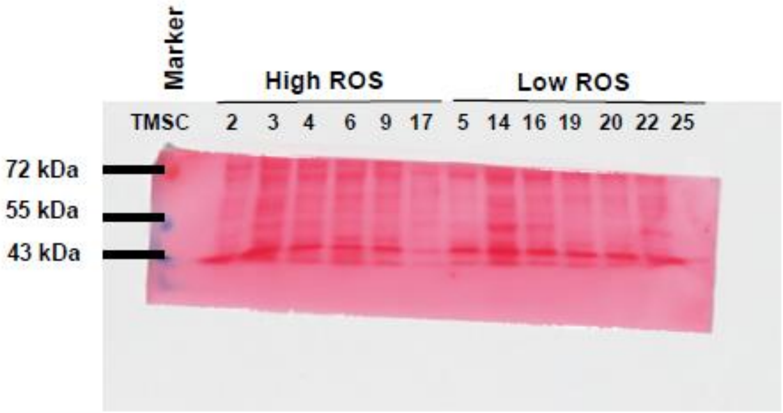

Supplementary Figure S2. Full Western Blot of Figure 3D,E.

Supplementary Table S1. Primers used in real-time PCR.

Gene	Primer sequence (5′-3′)	Reference
GAPDH	F: ACACCCACTCCTCCACCTTT	[1]
	R: TGCTGTAGCCAAATTCGTTG	
SFRP4	F: ACGAGCTGCCTGTCTATGAC	
	R: TGTCTGGTGTGATGTCTATCCAC	
MEDAG	F:TGAGCCTTTAATCCGAAGGA	
	R:TGATCTGGGTGACAGATTGC	

MFAP5	F:GGGTCAATAGTCAACGAGGAGA
	R:CTGTAGCGGGATCATTCACCA
PCOLCE2	F:GCAGTGAAGGTTTTCCTGGAGTG
	R:AGTCATAGCGGCACAGGTTGTC
APCDD1	F:CACAGTGCCATCACATGCTCA
	R:AGTGGCCCTCGATTGTAGGT
COL8A1	F:GGGAGTGCTGCTTACCATTTC
	R:AGCGGCTTGATCCCATAGTAG
PSG1	F:CTAACCCACCGGCACAGTAT
	R:AGAGCCCGCTATGCTTTGTA
PDPN	F:AACCAGCGAAGACCGCTATAA
	R:CGAATGCCTGTTACACTGTTGA
SNAP25	F:ACCAGTTGGCTGATGAGTCG
	R:CAAAGTCCTGATACCAGCATCTT
RUNX3	F:GCGAGGGAAGAGTTTCACCC
	R:TTGATGGCTCGGTGGTAGGT
PADI4	F:GGGGTGGTCGTGGATATTGC
	R:CCCGGTGAGGTAGAGTAGAGC
MAP2K2	F:CCAAGGTCGGCGAACTCAAA
	R:TCTCAAGGTGGATCAGCTTCC
MAPK3	F:CTACACGCAGTTGCAGTACAT
	R:CAGCAGGATCTGGATCTCCC
RAF1	F:CCGAACAAGCAAAGAACAGTG
	R:GACGCAGCATCAGTATTCCAAT
RAC2	F:CAACGCCTTTCCCGGAGAG
	R:TCCGTCTGTGGATAGGAGAGC
PTK2	F:CTTCAAACTGGTCAAATGCACTG
	R:CTCAGCCCATAGCACTCAGC
NOX1	F:GCACACCTGTTTAACTTTGACTG
	R:GGACTGGATGGGATTTAGCCA
NOX2	F:ACCGGGTTTATGATATTCCACCT
	R:GATTTCGACAGACTGGCAAGA
NOX3	F:CGTGGCGCATTTCTTCAACC
	R:GCTCTCGTTAGGGGTGTTGC
NOX4	F:CAGATGTTGGGGCTAGGATTG
	R:GAGTGTTCGGCACATGGGTA
NOX5	F:ACTCAGCAGTTTAAGACCATTGC
	R:GGACTCTTTCACATGCAGAGC
DUOX1	F:GCAGCGATTTGATGGGTGGTA
	R:AGGTGGGGTTCTCCCAAGG
DUOX2	F:ACGGTGTGTATCAGGCTCTG
	R:CACGTCGGAAAGAACATGGTAG

References

1. Ryu, K.H.; Kim, S.Y.; Kim, Y.R.; Woo, S.Y.; Sung, S.H.; Kim, H.S.; Jung, S.C.; Jo, I.; Park, J.W. Tonsil-derived mesenchymal stem cells alleviate concanavalin A-induced acute liver injury. *Exp Cell Res* **2014**, *326*, 143-154, doi:10.1016/j.yexcr.2014.06.007.
